# Dosimetric and Radiobiological Comparison of External Beam Radiotherapy Using Simultaneous Integrated Boost Technique for Esophageal Cancer in Different Location

**DOI:** 10.3389/fonc.2019.00674

**Published:** 2019-07-25

**Authors:** Lu Wang, Chengqiang Li, Xue Meng, Chengming Li, Xindong Sun, Dongping Shang, Linlin Pang, Yixiao Li, Jie Lu, Jinming Yu

**Affiliations:** ^1^Department of Radiation Oncology, School of Medicine, Shandong University, Jinan, China; ^2^Department of Radiation Oncology, Shandong Cancer Hospital and Institute, Shandong First Medical University and Shandong Academy of Medical Sciences, Jinan, China; ^3^Department of Radiation Physics, Shandong Cancer Hospital and Institute, Shandong First Medical University and Shandong Academy of Medical Sciences, Jinan, China; ^4^Department of Clinical Medicine, Jining Medical University, Jining, China

**Keywords:** esophageal cancer, IMRT, VMAT, HT, dosimetry, radiobiology, TCP, NTCP

## Abstract

**Objectives:** To compare treatment plans of intensity modulated radiotherapy (IMRT), volumetric modulated arc radiotherapy (VMAT), and helical tomotherapy (HT) with simultaneous integrated boost (SIB) technique for esophageal cancer (EC) of different locations using dosimetry and radiobiology.

**Methods:** Forty EC patients were planned for IMRT, VMAT, and HT plans, including 10 cases located in the cervix, upper, middle, and lower thorax, respectively. Dose-volume metrics, conformity index (CI), homogeneity index (HI), tumor control probability (TCP), and normal tissue complication probability (NTCP) were analyzed to evaluate treatment plans.

**Results:** HT showed significant improvement over IMRT and VMAT in terms of CI (*p* = 0.007), HI (*p* < 0.001), and TCP (*p* < 0.001) in cervical EC. IMRT yielded more superior CI, HI and TCP compared with VMAT and HT in upper and middle thoracic EC (all *p* < 0.05). Additionally, V30 (27.72 ± 8.67%), mean dose (1801.47 ± 989.58cGy), and NTCP (Niemierko model: 0.44 ± 0.55%; Lyman-Kutcher-Burman model: 0.61 ± 0.59%) of heart in IMRT were sharply reduced than VMAT and HT in middle thoracic EC. For lower thoracic EC, the three techniques offered similar CI and HI (all *p* > 0.05). But VMAT dramatically lowered liver V30 (9.97 ± 2.84%), and reduced NTCP of lungs (Niemierko model: 0.47 ± 0.48%; Lyman-Kutcher-Burman model: 1.41 ± 1.07%) and liver (Niemierko model: 0.10 ± 0.08%; Lyman-Kutcher-Burman model: 0.17 ± 0.17%).

**Conclusions:** HT was a good option for cervical EC with complex target coverage but little lungs and heart involvement as it achieved superior dose conformity and uniformity. Due to potentially improving tumor control and reducing heart dose with acceptable lungs sparing, IMRT was a preferred choice for upper and middle thoracic EC with large lungs involvement. VMAT could ameliorate therapeutic ratio and lower lungs and liver toxicity, which was beneficial for lower thoracic EC with little thoracic involvement but being closer to heart and liver. Individually choosing optimal technique for EC in different location will be warranted.

## Introduction

Esophageal cancer (EC) is the eighth most common carcinoma and the sixth leading cause of cancer-related death worldwide (1, 2). Radiotherapy (RT) plays a major role in multimodality management of EC. However, it still presents many challenges in treatment planning for EC. The planning target volume (PTV) is centralized and surround by several critical organs at risk (OARs) including lungs, heart, spinal cord, liver and kidneys. Although the esophagus can endure radiation dose up to 60 Gy, the highly radiosensitive nearby OARs must be spared to prevent potentially severe adverse events. More importantly, it is well-recognized that a superior RT technique which can facilitate the delivery of a substantial radiation dose to the tumor and avoid excess dose to the normal tissues in the tumor vicinity may improve patients' disappointing local control and survival (3, 4). Therefore, the question is raised that in order to improve target coverage and dose distributions of tumor as well as spare OARs, which is the optimal RT technique for EC?

Several modern external beam RT techniques are available currently to treat EC in clinical. Compared with 3-dimensional conformal radiotherapy (3D-CRT), intensity modulated radiotherapy (IMRT) has been introduced to improve target coverage (5, 6) and reduce the doses delivered to the surrounding normal tissues (7–9). However, even with IMRT, lungs and cardiac toxicities remain to be seriously regarded. Volumetric modulated arc radiotherapy (VMAT), regarded as a new generation linear-accelerator IMRT (10), can offer similar or even better dose distributions compared with IMRT and significantly shorten the delivery time (11–13). But the practical value of VMAT for EC is still debatable. Helical tomotherapy (HT) is an advanced image-guided radiotherapy technique (IGRT) combing daily pretreatment mega-voltage computed tomography (MVCT) scans with 360° dynamic rotational IMRT (14). Benefitting from pre-therapy visualization of target volumes and normal tissues, HT can further reduce irradiated OARs and permit dose escalation to tumor (15). Despite the advantages of HT have been performed in treatment of cervical cancer, nasopharyngeal carcinoma, and central nervous system tumors, its widespread application value in EC needs to be confirmed. In the past years, numerous studies have previously explored RT techniques for treating EC, but there is still no clear consensus on a preferred technique for it. Additionally, these studies have mainly focused on analysis of dose volume histogram (DVH) and dose distributions to evaluate RT plans. However, in some cases in which the treatment plans yield similar levels of dosimetric performance, analyzing other indices based on radiobiology may be essential when attempting to precisely evaluate the treatment outcome, particularly in terms of tumor control and normal tissue sparing (16, 17). But this issue is not received enough attention and the relevant data is rare. To the best of our knowledge, before our study, IMRT, VMAT, and HT treatment plans for patients with EC had not been analyzed based on both dosimetric and radiobiological parameters.

In this study, we aimed to not only compare the dose distributions to PTV and OARs for IMRT, VMAT, and HT, but also initially investigate the tumor control probability (TCP) and normal tissue complication probability (NTCP) of the three techniques. More importantly, we sought to select the optimal RT technique from IMRT, VMAT, and HT for EC in different location using both dosimetry and radiobiology.

## Materials and Methods

### Patients

Forty EC patients previously received definitive RT in our department were recruited for this study, involving 10 cases located in the cervix, upper, middle, and lower thorax, respectively. Each patient was re-planned planning for IMRT, VMAT, and HT. The clinicopathological data of patients was obtained from our hospital's medical records. All tumors were staged according to the 8th edition of the American Joint Committee on Cancer staging manual (18). Detailed baseline characteristics were shown in Table 1. Each patient gave written informed consent. This study was approved by Ethics Committee of Shandong Cancer Hospital and in accordance with the Declaration of Helsinki.

**Table 1:** Patients' characteristics.

**Characteristics**	**Median (range)**
Age (years)	65 (42–86)
Sex	
Male	31
Female	9
Length (cm)	4.6 (2–8)
Location	
Cervical	10
Upper	10
Middle	10
Lower	10
Histology	
Squamous	37
Non-squamous	3
TNM Stage	
II	10
III	30
Concurrent chemotherapy	
Yes	36
No	4

### Immobilization and Simulation

Patients were immobilized supinely by a thermoplastic custom-made mask on the head, neck and shoulders for the cervical, upper, and middle thoracic EC or with their arms raised above their heads using vacuum casts for the lower thoracic EC. Afterwards, the intravenous contrast-enhanced computed tomographic (CT) images of each patient for treatment planning were obtained. These images were taken at a 3 mm thickness throughout the neck, thorax, and upper abdomen that enlarge to 10 cm beyond the tumor's border. The simulation CT images were transferred to the Eclipse system (Varian Medical Systems, Palo Alto, CA, Version 13.5.35) for IMRT and VMAT planning. After delineating the targets and OARs, the CT datasets were transmitted to the TomoTherapy® Planning Station (Accuray, Sunnyvale, CA, Hi-Art, Version 5.1.3).

### Target Volumes and OARs Delineation

The delineation of target volumes and OARs referred to the Radiotherapy and Oncology Group (RTOG) guidelines. The gross tumor volume (GTV) involving the primary tumor and positive lymph nodes were identified by the diagnostic CT scans, positron emission tomography/CT(PET-CT) scans, barium swallow, and esophagoscopy. The clinical target volume high (CTV_H_) was defined as 3–4 cm superior-inferior margins and 1 cm radial margins with respect to the GTV with 1 cm uniform margins for positive nodes. The elective nodal regions depending on the location of the primary tumor were included in the clinical target volume low (CTV_L_). The planning target volume high/low (PTV_H/L_) was delineated with additional 0.5–1.0 cm margins to the CTV_H/L_. All patients were treated by simultaneous integrated boost (SIB) technique. The prescribed dose was 50.4 Gy in 28 fractions for the PTV_L_ and 59.36 Gy in 28 fractions for the PTV_H_ according to Welsh et al. (19). The lungs, heart, spinal cord, and liver were contoured as the dose-limiting OARs. In detail, all inflated and collapsed, fibrotic, and emphysematic lungs were contoured with inclusion of small vessels extending beyond the hilar regions, excluding the proximal bronchial tree. The contour of heart was along with the pericardial sac. The superior aspect (or base) began at the level of the inferior aspect of the pulmonary artery passing the midline and extended inferiorly to the apex of the heart. The spinal cord was delineated starting at the same cranial level as the esophagus to the bottom of L2 or at the level in which the cord ended. For liver contouring, gallbladder should be excluded. The inferior vena cava (IVC) was not included when it was discrete from the liver. The portal vein (PV) should be included in the liver contour when caudate lobe was seen to the left of PV, but excluded when caudate lobe was seen to the posterior of PV (20, 21).

### Treatment Planning and Delivery

IMRT and VMAT plans were generated on a Varian treatment planning system (TPS). They were delivered with 6 MV photons beams from a Varian trilogy linear accelerator equipped with a Millennium Multileaf Collimator (MLC) with 120 leaves (spatial resolution of 5 mm at isocenter for the central 20 cm and of 10 mm in the outer 2 × 10 cm, a maximum leaf speed of 2.5 cm/s and a leaf transmission of ~1.5%). The dose calculations were performed by the anisotropic analytical algorithm (AAA) and a grid resolution of 2.5 mm, considering heterogeneity corrections. IMRT plans were implemented using 7, 9, or 10 coplanar fields. The arrangement of each beam was optimized for PTV coverage and OARs sparing. Specifically, for cervical EC, 9 coplanar fields were chosen. Because of the extremely irregular shape of tumor, 10 coplanar fields in only two cases were created by adding an additional field for tumor target to 9 coplanar fields in order to achieve better target conformity. Seven coplanar fields were selected for upper, middle and lower thoracic EC. Beam geometry consisted of each treatment field with the following gantry angles: 0°/35°/70°/160°/200°/290°/325° (7 fields), 0°/35°/70°/160°/175°/200°/230°/290°/325° (9 fields), and 0°/35°/70°/160°/175°/200°/230°/290°/325° with an additional gantry angle based on individual irregular shape of tumor (10 fields). The MLC leaf sequences were generated using the dynamic sliding window IMRT delivery technique with a fixed dose rate (DR) of 400 MU/min. For VMAT plans, since each single arc was limited to a sequence of 177 control points, the application of two coplanar arcs that increase the modulation factor during optimization, could allow the optimizer to achieve a higher target homogeneity and lower OARs involvement at the same time (11, 22). Thus, VMAT plans were generated by two coplanar arcs of 360° with opposition rotation (clock-wise or counter clock-wise; without the sectors from 80 to 110° and 250 to 280° aiming for sparing lungs; a collimator rotation between ±30°). Dynamic MLC, changeable instantaneous DR and variable gantry rotational speed were utilized to optimize the dose distributions of VMAT. The maximum DR of VMAT was fixed at 600 MU/min. HT plans were designed on a tomotherapy planning station. They were optimized by superposition convolution algorithm with a dose calculation grid resolution of 1.875 × 1.875 mm^2^. A field width of 2.51 cm, a pitch of 0.287, and a modulation factor of 3.00 were implemented in our study. HT delivered 6 MV X-ray beams with a 64 leaves binary MLC of a 40 cm wide fan of thicknesses 0.5–5.0 cm to an isocenter 85 cm away from the source. The DR of HT was set to 846 MU/min. Previous study reported when more beams from different directions focused on the tumor, the volume of normal tissue with low-dose exposure would increase (23). HT with 51 gantry angles per rotation 360° led to extensive low-dose distribution in lungs during RT, especially for EC. Consequently, the fan-shaped virtual blocks were used in all HT plans to restrict beamlets and optimize dose distribution to spare lungs.

### Treatment Plan Evaluation

For PTVs, D2, D98, D50, Dmean, conformity index (CI), and homogeneity index (HI) were analyzed. The CI was defined as: CI = (TV_PV_/V_PTV_)/(V_TV_/TV_PV_), where V_PTV_ was the volume of the PTV, TV_PV_ was the volume of PTV covered by the prescribed isodose line, V_TV_ was the volume enclosed by the prescription dose line (24). The 95% isodose was chosen as the prescription isodose line. The value of CI varied from 0 to 1. The value was closer to 1, the conformity of dose distribution was better. The HI was defined as: HI = (D2–D98)/D50, where D2, D50, and D98 were the dose at 2, 50, and 98% of the PTV, respectively (25). A lower HI indicated higher dose homogeneity. For OARs, mean lung dose (MLD) and the volumes of lung receiving dose at least 5, 10, 20, and 30Gy (V5, V10, V20, and V30), mean heart dose (MHD) and the volumes of heart receiving dose at least 30, 40, and 50Gy (V30, V40, and V50), the volumes of liver receiving dose at least 20 and 30 Gy (V20 and V30), and the maximum dose (Dmax) to the spinal cord were analyzed.

Radiobiological parameters including TCP and NTCP were evaluated using Niemierko's phenomenological model (26–28). The calculations were as follows. The physical dose was firstly converted to the biologically equivalent dose of 2 Gy (EQD_2_) by the linear-quadratic (LQ) model and then was used to calculate the equivalent uniform dose (EUD). The TCP and NTCP were finally obtained based on the EUD. The parameters and endpoints for TCP and NTCP calculations were taken from previous studies (17, 29, 30) and were shown in Table 2A. More importantly, it was well-known that the parameters of the radiobiological models would influence the results, therefore, we also used the Webb-Nahum model (WN model) and the Lyman-Kutcher-Burman (LKB) model to further confirm our results of TCP and NTCP calculations. The WN model calculated the predictive TCP from the DVH for the PTV on basis of a normal distribution of radiosensitivity α values among a cohort of patients. The parameters used in this model were α_m_ = 0.40 Gy^−1^, σ_α_ = 0.08 Gy^−1^, and ρ = 10^7^/cm^3^, where α_m_ and σ_α_ were the mean and standard deviation of the values of α, respectively, ρ represented the uniform clonogenic cell density (31, 32). The LKB model has been recognized to predict the NTCP values of OARs (33, 34). The tolerance dose for a 5% (TD_5_) or 50% (TD_50_) complication, n and m values in this model for predicting complications of OARs were obtained from Burman et al. and were displayed in Table 2B (35). All DVH data were exported from TPS and imported into MATLAB 2017a (Mathworks, Natick, MA, USA) to calculate TCP and NTCP.

**Table 2:** Radiobiological parameters used to calculate TCP and NTCP by Niemierko's model **(A)** or LKB model **(B)**.

**Tissue**	**Volume type/endpoint**	**TD_**50**_/TCD_**50**_ (Gy)**	**γ_**50**_**	***a***	***α/β*(Gy)**
**(A)**					
Esophagus (PTV)	Tumor	TCD_50_ = 49.09	2.16	−13	10
Lung	OAR: symptomatic pneumonitis	TD_50_ = 24.50	2	1	3
Heart	OAR: pericarditis	TD_50_ = 48.00	3	3	2
Spinal cord	OAR: myelitis/necrosis	TD_50_ = 66.50	4	13	2
Liver	OAR: radiation induced liver disease	TD_50_ = 40.00	3	2	1.5
**Tissue**	**Volume type/endpoint**	**TD**_**50**_ **(Gy)**	**TD**_**5**_ **(Gy)**	**Size factor (n)**	**Slope (m)**
**(B)**					
Lung	OAR: grade≥2 pneumonitis	24.50	17.50	0.87	0.18
Heart	OAR: pericarditis	48.00	40.00	0.35	0.10
Spinal cord	OAR: myelitis/necrosis	66.50	47.00	0.05	0.175
Liver	OAR: radiation induced liver disease	40.00	30.00	0.32	0.15

*TCP, tumor control probability; NTCP, normal tissue complication probability; PTV, planning target volume; OAR, organ at risk; LKB model, Lyman-Kutcher-Burman model*.

Plans were normalized to 95% of the PTV received 100% of the prescribed dose. The dose constraints were defined for OARs as follows: spinal cord < 50 Gy; total lungs: V5 < 60%, V20 < 30%, V30 < 20%, MLD < 20 Gy; heart: V30 ≤ 4 0%, V40 < 3 0%; liver: V20 ≤ 30%, V30 ≤ 20%. To ensure the tumor coverage requirements, a waiver could be applied for these dose constraints in some cases.

### Statistical Analyses

The data was expressed as mean ± standard deviation or median (range). ANOVA and *post-hoc* two-tailed paired *t*-tests were used. *P* < 0.05 indicated statistically significant. All data were performed with the Statistical Package for Social Science program (SPSS for Windows, version 17.0, SPSS Inc., Chicago, IL).

## Results

**Figure 1:**
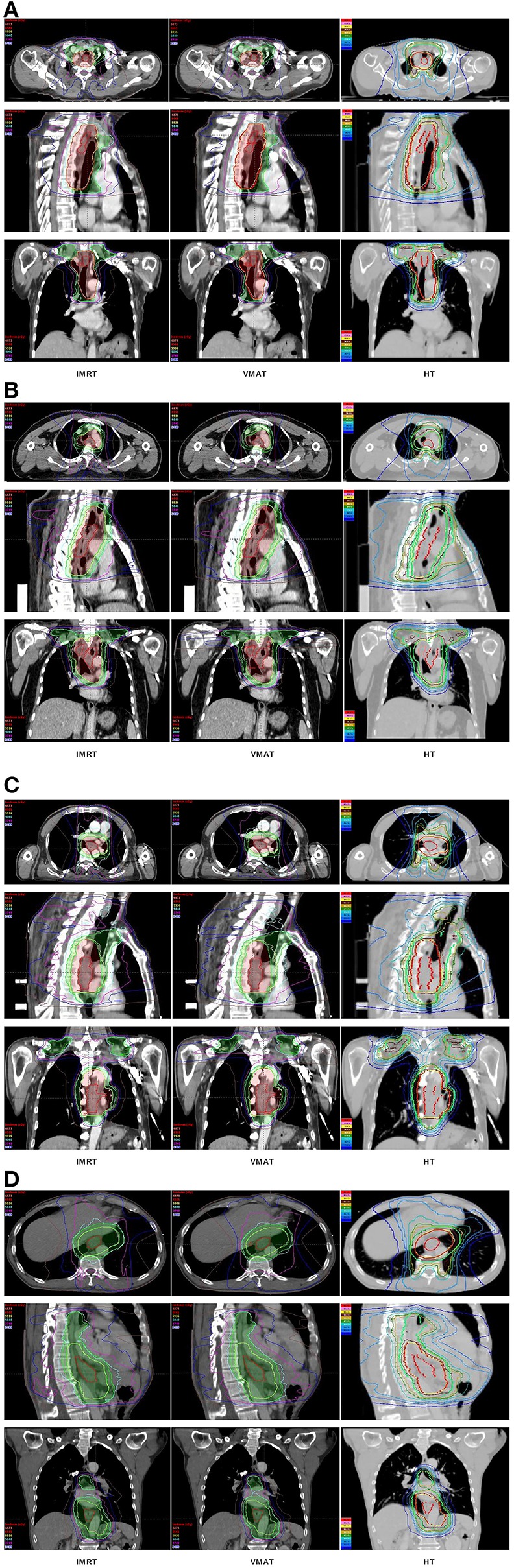
Dose distributions. Dose distributions of IMRT (left), VMAT (middle), HT (right) for a cervical **(A)**, upper **(B)**, middle **(C)**, and lower **(D)** thoracic EC in axial, sagittal, and coronal views. EC, esophageal cancer; IMRT, intensity modulated radiotherapy; VMAT, volumetric modulated arc radiotherapy; HT, helical tomotherapy.

**Figure 2:**
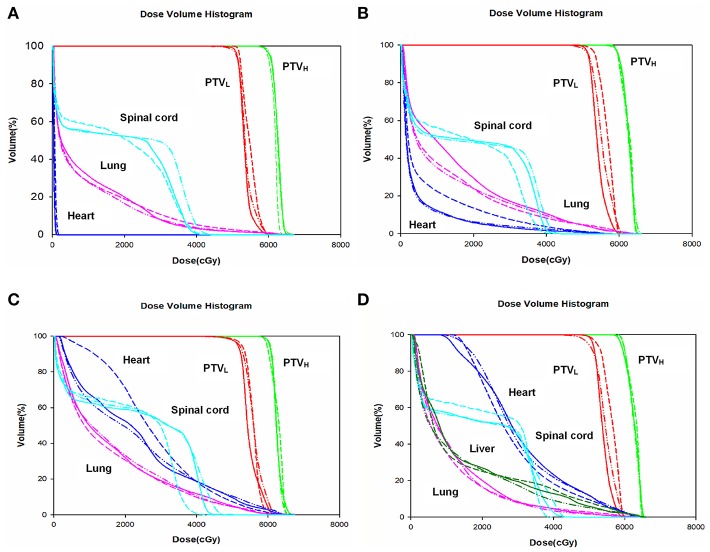
Dose volume histogram. Comparing the dose volume histogram from IMRT, VMAT, and HT of a patient with cervical **(A)**, upper **(B)**, middle **(C)**, and lower **(D)** thoracic EC. EC, esophageal cancer. Solid line: IMRT; Dashed line: VMAT; Dash-dot-dotted line: HT. IMRT, intensity modulated radiation therapy; VMAT, volumetric modulated radiation therapy; HT, helical tomotherapy. Green: PTV_H_; Red: PTV_L_; Cyan: spinal cord; Pink: lung; Blue: heart; Dark green: liver.

The dose-volume parameters of PTVs were listed in Table 3. In cervical EC, HT yielded more superior HI and CI than IMRT and VMAT for PTV_H_ (HI: 0.10 ± 0.01, 0.11 ± 0.02, 0.07 ± 0.03, respectively, *p* < 0.001; CI: 0.84 ± 0.07, 0.81 ± 0.05, 0.90 ± 0.06, respectively, *p* = 0.007). Meanwhile, the HI of PTV_L_ in HT showed more appropriate dose homogeneity (0.16 ± 0.03, 0.19 ± 0.03, 0.12 ± 0.04, respectively; *p* = 0.001). D2, D98, D50, and Dmean of PTV_H_ as well as D98 of PTV_L_ were significantly different in IMRT, VMAT, and HT (all *p* < 0.05). In upper thoracic EC, the best HI (0.09 ± 0.01, 0.14 ± 0.03) and CI (0.88 ± 0.04, 0.72 ± 0.07) of PTV_H_ and PTV_L_ were generated by IMRT rather than VMAT and HT (all *p* < 0.05). Moreover, IMRT, VMAT, and HT provided the obviously diverse results in terms of D2 and D98 of PTV_H_ (all *p* < 0.05), as well as D50, D98, and Dmean of PTV_L_ (all *p* < 0.05). For middle thoracic EC, the HI (0.07 ± 0.01) and CI (0.87 ± 0.05) of PTV_H_ and HI (0.14 ± 0.02) of PTV_L_ in IMRT were significant improvement over VMAT and HT (all *p* < 0.05). Besides, the three techniques generated different levels of D2 and D98 for PTV_H_ and D2, D98, D50, and Dmean for PTV_L_ (all *p* < 0.05). In lower thoracic EC, no significant differences were observed for the three plans regarding HI and CI of PTV_H_ and PTV_L_ (all *p* > 0.05). The detailed information of the treatment plans is shown in the Supplementary Material, which could be available online. Figures 1A–D showed the dose distributions of IMRT, VMAT, and HT plans in a patient with EC in different location. Figures 2A–D displayed the DVHs for the 3 plans in the same case.

**Table 3:** Dosimetric results for PTVs in cervical, upper, middle, and lower thoracic EC.

	**IMRT**	**VMAT**	**HT**	***p*-value^⋆^**	***p*****-value^Δ^**		
					**I VS. V**	**I VS. T**	**V VS. T**
**PTV**_**H**_							
**D2(cGy)**							
Cervical	6478.46 ± 96.23	6526.78 ± 48.85	6335.81 ± 77.24	<0.001	0.170	<0.001	<0.001
Upper	6507.76 ± 38.24	6533.05 ± 45.74	6418.53 ± 85.45	0.001	0.356	0.003	<0.001
Middle	6371.68 ± 64.43	6498.71 ± 43.59	6443.21 ± 85.98	0.001	<0.001	0.024	0.075
Lower	6525.30 ± 35.45	6506.72 ± 31.69	6466.89 ± 83.34	0.072	0.460	0.026	0.119
**D98(cGy)**							
Cervical	5816.89 ± 97.47	5861.07 ± 66.96	5936.70 ± 24.50	0.002	0.168	0.001	0.022
Upper	5918.25 ± 43.53	5849.42 ± 59.73	5789.34 ± 78.84	<0.001	0.020	<0.001	0.040
Middle	5983.38 ± 43.43	5925.98 ± 70.25	5906.07 ± 49.95	0.012	0.029	0.004	0.431
Lower	5878.93 ± 60.86	5932.87 ± 78.32	5943.08 ± 19.82	0.045	0.049	0.021	0.699
**D50(cGy)**							
Cervical	6231.36 ± 85.52	6265.35 ± 11.07	6162.65 ± 86.22	0.010	0.290	0.038	0.003
Upper	6241.84 ± 40.12	6249.61 ± 38.90	6213.42 ± 49.18	0.160	0.689	0.151	0.071
Middle	6246.68 ± 36.35	6267.16 ± 23.37	6239.52 ± 45.85	0.229	0.219	0.664	0.101
Lower	6261.71 ± 16.48	6236.25 ± 80.05	6246.40 ± 64.40	0.639	0.352	0.573	0.708
**Dmean(cGy)**							
Cervical	6217.16 ± 98.79	6248.40 ± 0.00	6160.84 ± 57.24	0.020	0.299	0.067	0.006
Upper	6248.40 ± 0.00	6251.76 ± 10.63	6222.67 ± 64.17	0.185	0.843	0.137	0.095
Middle	6248.40 ± 0.00	6251.66 ± 10.31	6224.20 ± 53.94	0.126	0.820	0.099	0.063
Lower	6172.45 ± 53.17	6249.73 ± 4.17	6188.40 ± 78.18	0.252	0.118	0.742	0.211
**HI**							
Cervical	0.10 ± 0.01	0.11 ± 0.02	0.07 ± 0.03	<0.001	0.641	<0.001	<0.001
Upper	0.09 ± 0.01	0.11 ± 0.01	0.11 ± 0.01	0.016	0.007	0.022	0.632
Middle	0.07 ± 0.01	0.09 ± 0.01	0.08 ± 0.02	0.028	0.011	0.041	0.571
Lower	0.10 ± 0.01	0.08 ± 0.02	0.09 ± 0.01	0.077	0.027	0.143	0.410
**CI**							
Cervical	0.84 ± 0.07	0.81 ± 0.05	0.90 ± 0.06	0.007	0.256	0.033	0.002
Upper	0.88 ± 0.04	0.83 ± 0.05	0.78 ± 0.06	0.001	0.031	<0.001	0.077
Middle	0.87 ± 0.05	0.78 ± 0.09	0.79 ± 0.06	0.027	0.013	0.030	0.714
Lower	0.85 ± 0.10	0.83 ± 0.10	0.82 ± 0.11	0.875	0.814	0.608	0.781
**PTV**_**L**_							
**D2(cGy)**							
Cervical	5861.65 ± 133.40	5967.33 ± 101.46	5869.77 ± 155.96	0.158	0.085	0.892	0.110
Upper	5913.69 ± 110.09	5984.71 ± 116.95	5968.32 ± 112.76	0.357	0.174	0.292	0.750
Middle	5921.33 ± 73.99	6066.44 ± 184.79	5919.89 ± 73.63	0.017	0.013	0.979	0.013
Lower	6072.06 ± 220.04	6035.84 ± 155.95	5964.20 ± 109.36	0.358	0.634	0.163	0.349
**D98(cGy)**							
Cervical	5048.98 ± 129.40	4948.75 ± 83.57	5165.69 ± 98.86	<0.001	0.043	0.020	<0.001
Upper	5178.90 ± 93.80	4979.70 ± 83.58	5077.99 ± 93.78	<0.001	<0.001	0.019	0.022
Middle	5155.66 ± 145.66	4962.82 ± 143.84	5076.86 ± 122.13	0.015	0.004	0.211	0.075
Lower	5043.85 ± 95.80	4969.51 ± 215.30	5108.15 ± 81.08	0.201	0.406	0.326	0.076
**D50(cGy)**							
Cervical	5348.64 ± 172.41	5436.82 ± 119.07	5453.10 ± 139.41	0.242	0.186	0.120	0.804
Upper	5419.62 ± 86.35	5501.09 ± 165.49	5588.11 ± 110.96	0.020	0.158	0.006	0.132
Middle	5469.51 ± 68.04	5643.71 ± 222.50	5619.79 ± 116.61	0.031	0.015	0.034	0.725
Lower	5566.05 ± 188.00	5509.18 ± 253.57	5609.34 ± 165.13	0.558	0.542	0.642	0.286
**Dmean(cGy)**							
Cervical	5395.53 ± 120.16	5449.55 ± 100.28	5470.70 ± 116.65	0.322	0.293	0.147	0.678
Upper	5435.71 ± 83.75	5503.41 ± 138.66	5600.40 ± 81.24	0.006	0.159	0.002	0.048
Middle	5479.00 ± 57.18	5617.52 ± 175.71	5600.40 ± 73.17	0.023	0.012	0.025	0.741
Lower	5607.75 ± 286.29	5560.85 ± 108.07	5594.60 ± 137.28	0.856	0.593	0.880	0.700
**HI**							
Cervical	0.16 ± 0.03	0.19 ± 0.03	0.12 ± 0.04	0.001	0.036	0.036	<0.001
Upper	0.14 ± 0.03	0.18 ± 0.03	0.16 ± 0.02	0.003	0.001	0.036	0.138
Middle	0.14 ± 0.02	0.19 ± 0.03	0.17 ± 0.03	0.001	<0.001	0.024	0.081
Lower	0.19 ± 0.03	0.19 ± 0.04	0.16 ± 0.03	0.127	0.951	0.083	0.074
**CI**							
Cervical	0.65 ± 0.06	0.66 ± 0.04	0.68 ± 0.07	0.457	0.839	0.245	0.335
Upper	0.72 ± 0.07	0.67 ± 0.05	0.62 ± 0.05	0.001	0.039	<0.001	0.059
Middle	0.68 ± 0.06	0.69 ± 0.05	0.68 ± 0.09	0.899	0.751	0.899	0.657
Lower	0.74 ± 0.05	0.72 ± 0.07	0.70 ± 0.09	0.338	0.468	0.144	0.450

*EC, esophageal cancer; PTV_H/L_, planning target volume high/low; IMRT(I), intensity modulated radiotherapy; VMAT(V), volumetric modulated arc radiotherapy; HT, helical tomotherapy; CI, conformity index; HI, homogeneity index*.

⋆ *ANOVA*;

Δ *post-hoc two-tailed paired t-tests*.

The dosimetric results for lungs, heart, spinal cord and liver were summarized in Table 4. Specifically, V5, V10, V20, V30, and MLD of lungs showed no significant difference in any EC position in IMRT, VMAT, and HT (all *p* > 0.05). However, we surprisedly found the heart V30 and MHD of IMRT in middle thoracic EC were sharply reduced than that of VMAT and HT (all *p* < 0.05). The Dmax to spinal cord in HT displayed the lowest level compared with IMRT and VMAT in EC of all locations (all *p* < 0.05). Furthermore, we investigated that the liver V30 of VMAT (9.97 ± 2.84%) in lower thoracic EC was dramatically decreased than two other plans (15.24 ± 5.48%, 17.04 ± 6.57%, *p* = 0.014).

**Table 4:** Dosimetric results for OARs in cervical, upper, middle, and lower thoracic EC.

	**IMRT**	**VMAT**	**HT**	***p-*value^⋆^**	***p*****-value^Δ^**		
					**I VS. V**	**I VS. T**	**V VS. T**
**Total lungs**							
**V5(%)**							
Cervical	31.64 ± 8.02	30.93 ± 9.51	34.74 ± 8.76	0.593	0.858	0.437	0.340
Upper	54.53 ± 12.14	53.35 ± 13.90	56.04 ± 11.64	0.893	0.836	0.791	0.638
Middle	56.63 ± 13.16	58.04 ± 10.42	59.11 ± 7.38	0.871	0.768	0.604	0.823
Lower	58.96 ± 9.87	56.42 ± 9.21	56.61 ± 8.64	0.792	0.544	0.574	0.964
**V10(%)**							
Cervical	22.57 ± 5.89	22.59 ± 7.02	25.45 ± 6.43	0.525	0.995	0.327	0.331
Upper	39.03 ± 10.12	39.13 ± 11.31	42.18 ± 8.91	0.735	0.982	0.493	0.507
Middle	41.25 ± 11.34	43.21 ± 9.35	43.23 ± 5.97	0.857	0.635	0.633	0.997
Lower	42.66 ± 7.45	42.17 ± 7.87	41.08 ± 7.69	0.895	0.888	0.649	0.753
**V20(%)**							
Cervical	15.07 ± 4.41	14.04 ± 4.54	15.56 ± 4.30	0.737	0.609	0.803	0.448
Upper	27.80 ± 8.13	24.97 ± 8.72	26.08 ± 6.27	0.717	0.422	0.625	0.751
Middle	27.71 ± 8.46	26.14 ± 8.25	25.13 ± 5.76	0.748	0.646	0.454	0.770
Lower	27.00 ± 5.44	23.01 ± 5.84	20.84 ± 5.15	0.055	0.116	0.018	0.384
**V30(%)**							
Cervical	9.09 ± 3.47	8.06 ± 2.54	9.31 ± 2.51	0.592	0.432	0.865	0.341
Upper	16.66 ± 5.86	14.35 ± 5.26	14.87 ± 3.55	0.561	0.309	0.428	0.819
Middle	16.12 ± 6.42	13.49 ± 6.26	13.67 ± 4.69	0.541	0.325	0.357	0.947
Lower	12.30 ± 3.61	10.60 ± 4.20	10.75 ± 3.69	0.556	0.330	0.376	0.928
**MLD(cGy)**							
Cervical	805.71 ± 210.58	774.72 ± 220.37	871.40 ± 218.20	0.600	0.751	0.503	0.327
Upper	1356.68 ± 343.12	1284.92 ± 349.69	1368.30 ± 279.70	0.826	0.626	0.937	0.572
Middle	1370.31 ± 362.93	1329.26 ± 323.52	1354.70 ± 242.74	0.957	0.772	0.912	0.857
Lower	1391.64 ± 261.44	1261.39 ± 231.31	1256.50 ± 229.22	0.377	0.238	0.221	0.964
**Heart**							
**V30(%)**							
Cervical	—	—	—	—	—	—	—
Upper	16.79 ± 19.98	16.44 ± 21.39	19.73 ± 18.78	0.923	0.969	0.746	0.717
Middle	27.72 ± 8.67	37.51 ± 11.12	40.21 ± 9.76	0.022	0.036	0.009	0.547
Lower	37.02 ± 11.48	36.02 ± 12.21	36.81 ± 2.55	0.976	0.836	0.966	0.870
**V40(%)**							
Cervical	—	—	—	—	—	—	—
Upper	9.97 ± 12.49	10.20 ± 14.05	9.63 ± 9.92	0.994	0.968	0.950	0.918
Middle	18.10 ± 8.51	21.10 ± 9.63	17.53 ± 5.14	0.570	0.410	0.874	0.327
Lower	19.06 ± 6.82	19.30 ± 6.51	17.43 ± 4.21	0.748	0.927	0.546	0.487
**V50(%)**							
Cervical	—	—	—	—	—	—	—
Upper	4.62 ± 6.36	5.44 ± 7.63	4.55 ± 5.61	0.945	0.782	0.981	0.764
Middle	8.35 ± 5.33	9.70 ± 6.13	7.30 ± 3.29	0.574	0.556	0.645	0.298
Lower	9.31 ± 4.68	9.64 ± 4.41	7.85 ± 3.11	0.595	0.860	0.437	0.342
**MHD(cGy)**							
Cervical	196.70 ± 309.77	190.07 ± 271.79	294.50 ± 408.84	0.740	0.965	0.520	0.492
Upper	1251.10 ± 1163.32	1249.30 ± 1195.94	1554.80 ± 1128.37	0.797	0.997	0.564	0.562
Middle	1801.47 ± 989.58	2575.78 ± 675.62	2784.18 ± 588.21	0.020	0.033	0.008	0.550
Lower	2664.21 ± 591.19	2647.24 ± 590.00	2819.50 ± 404.44	0.733	0.944	0.522	0.478
**Spinal Cord**							
**Max dose(cGy)**							
Cervical	4534.48 ± 243.89	4541.27 ± 272.63	4253.75 ± 154.26	0.013	0.948	0.011	0.009
Upper	4729.85 ± 271.21	4782.45 ± 227.84	4219.50 ± 168.94	<0.001	0.608	<0.001	<0.001
Middle	4589.54 ± 242.58	4656.57 ± 322.64	4272.10 ± 229.59	0.008	0.581	0.013	0.003
Lower	4479.68 ± 152.19	4408.64 ± 290.11	4174.40 ± 250.15	0.020	0.510	0.008	0.036
**Liver**							
**V20(%)**							
Lower	21.92 ± 7.06	19.97 ± 7.05	24.76 ± 7.47	0.341	0.551	0.385	0.148
**V30(%)**							
Lower	15.24 ± 5.48	9.97 ± 2.84	17.04 ± 6.57	0.014	0.032	0.444	0.005

*OARs, organs at risk; EC, esophageal cancer; IMRT(I), intensity modulated radiotherapy; VMAT(V), volumetric modulated arc radiotherapy; HT, helical tomotherapy; MLD, mean lung dose; MHD, mean heart dose*.

⋆ *ANOVA*;

Δ *post-hoc two-tailed paired t-tests*.

The data of TCP and NTCP was listed in Table 5. Although three different methods including Niemierko's phenomenological model, WN model and LKB model were utilized to calculate TCP and NTCP, the similar results were still observed. In detail, HT yielded improved TCP over IMRT and VMAT in cervical EC (*p* < 0.01). The TCP of IMRT in upper and middle thoracic EC were both notably increased in comparison with VMAT and HT (all *p* < 0.05). But for lower thoracic EC, VMAT showed better TCP followed by HT, and IMRT had the poorest result. Additionally, we found that NTCP of heart could be dramatically reduced by IMRT rather than VMAT and HT in middle thoracic EC (*p* < 0.05). VMAT significantly lowered NTCP of lungs and liver in lower thoracic EC compared to IMRT and HT (all *p* < 0.05). Besides, the different trends of NTCP of heart and lungs were not observed in other positions. NTCP of spinal cord was extremely low in all cases.

**Table 5:** The results of TCP for EC and NTCP for OARs.

	**IMRT**	**VMAT**	**HT**	***p*–value^⋆^**	***p*****–value^Δ^**		
					**I VS. V**	**I VS. T**	**V VS. T**
**Esophageal TCP(%)**							
**Niemierko model**							
Cervical	83.71 ± 1.57	84.28 ± 2.50	87.99 ± 1.94	<0.001	0.537	<0.001	<0.001
Upper	87.40 ± 2.48	84.39 ± 2.74	85.04 ± 2.28	0.030	0.012	0.045	0.569
Middle	87.24 ± 2.31	85.10 ± 2.41	84.49 ± 1.87	0.024	0.039	0.010	0.538
Lower	84.21 ± 3.07	88.05 ± 1.96	85.56 ± 1.61	0.003	0.001	0.198	0.022
**WN model**							
Cervical	85.45 ± 0.97	85.66 ± 1.82	88.57 ± 1.36	<0.001	0.746	<0.001	<0.001
Upper	88.24 ± 1.71	85.49 ± 1.92	86.30 ± 1.91	0.008	0.003	0.027	0.331
Middle	89.11 ± 0.38	87.61 ± 1.20	86.53 ± 1.25	<0.001	0.003	<0.001	0.027
Lower	85.31 ± 2.49	88.43 ± 1.36	85.78 ± 2.15	0.004	0.002	0.616	0.008
**Lung NTCP(%)**							
**Niemierko model**							
Cervical	0.01 ± 0.02	0.02 ± 0.03	0.02 ± 0.02	0.973	0.817	0.885	0.931
Upper	0.35 ± 0.69	0.25 ± 0.50	0.38 ± 0.60	0.887	0.719	0.919	0.645
Middle	0.62 ± 0.88	0.47 ± 0.62	0.59 ± 0.71	0.886	0.646	0.931	0.709
Lower	1.43 ± 0.95	0.47 ± 0.48	1.49 ± 0.92	0.014	0.014	0.860	0.009
**LKB model**							
Cervical	0.50 ± 0.29	0.47 ± 0.30	0.49 ± 0.31	0.969	0.813	0.959	0.853
Upper	1.50 ± 0.66	1.46 ± 0.96	1.64 ± 0.91	0.880	0.915	0.710	0.633
Middle	2.96 ± 2.08	2.62 ± 1.61	2.79 ± 1.56	0.915	0.677	0.831	0.838
Lower	2.43 ± 1.09	1.41 ± 1.07	2.59 ± 0.99	0.038	0.039	0.733	0.018
**Heart NTCP(%)**							
**Niemierko model**							
Cervical	0	0	0	—	—	—	—
Upper	0.24 ± 0.48	0.46 ± 0.95	0.64 ± 1.04	0.588	0.574	0.308	0.642
Middle	0.44 ± 0.55	1.42 ± 0.98	1.44 ± 0.96	0.021	0.016	0.014	0.970
Lower	0.28 ± 0.31	0.65 ± 0.60	0.47 ± 0.39	0.204	0.078	0.356	0.378
**LKB model**							
Cervical	0	0	0	—	—	—	—
Upper	0.53 ± 0.89	0.61 ± 1.06	0.66 ± 1.03	0.953	0.848	0.761	0.910
Middle	0.61 ± 0.59	1.61 ± 1.00	1.63 ± 0.98	0.023	0.017	0.015	0.961
Lower	1.53 ± 0.88	1.70 ± 1.01	1.73 ± 1.01	0.885	0.700	0.648	0.944
**Spinal Cord NTCP(%)**							
**Niemierko model**							
Cervical	0	0	0	—	—	—	—
Upper	(0.07 ± 0.11) × 10^−2^	(0.07 ± 0.07) × 10^−2^	(0.07 ± 0.21) × 10^−2^	0.990	0.903	0.903	1.000
Middle	(0.02 ± 0.02) × 10^−2^	(0.03 ± 0.03) × 10^−2^	(0.02 ± 0.01) × 10^−2^	0.271	0.307	0.557	0.113
Lower	(0.01 ± 0.03) × 10^−2^	(0.01 ± 0.02) × 10^−2^	(0.01 ± 0.01) × 10^−2^	0.717	1.000	0.483	0.483
**LKB model**							
Cervical	0	0	0	—	—	—	—
Upper	(0.69 ± 0.53) × 10^−2^	(0.79 ± 0.61) × 10^−2^	(0.40 ± 0.35) × 10^−2^	0.224	0.664	0.214	0.098
Middle	(0.53 ± 0.56) × 10^−2^	(0.86 ± 1.13) × 10^−2^	(0.44 ± 0.58) × 10^−2^	0.475	0.364	0.803	0.250
Lower	(1.66 ± 2.60) × 10^−2^	(1.43 ± 2.48) × 10^−2^	(0.54 ± 1.06) × 10^−2^	0.482	0.814	0.257	0.365
**Liver NTCP(%)**							
**Niemierko model**							
Lower	0.57 ± 0.28	0.10 ± 0.08	0.73 ± 0.29	<0.001	<0.001	0.138	<0.001
**LKB model**							
Lower	0.66 ± 0.42	0.17 ± 0.17	0.79 ± 0.42	0.001	0.005	0.422	0.001

*EC, esophageal cancer; TCP, tumor control probability; NTCP, normal tissue complication probability; OARs, organs at risk; WN model, Webb-Nahum model; LKB model, Lyman-Kutcher-Burman model; IMRT(I), intensity modulated radiotherapy; VMAT(V), volumetric modulated arc radiotherapy; HT, helical tomotherapy*.

⋆ *ANOVA*;

Δ *post-hoc two-tailed paired t-tests*.

## Discussion

New and innovative RT techniques are urgently needed to deliver more safely and accurately for treating EC. Based on anatomical location, the esophagus is divided into four parts with unique characteristics. The cervical area frequently yields complex target coverage but implicating relatively small lungs volume. While, larger lungs volume is involved in the thoracic area. The lower area has smaller target volume but being closer to heart, liver, and kidneys. Notably, the primary tumor location is absolutely vital in deciding the optimal RT technique with regard to achieving an acceptable tradeoff between radiation induced toxicity and target coverage. Although increasing evidences have demonstrated that RT techniques could display different advantages and disadvantages for EC in different locations, the results were still inconclusive and needed to be further confirmed. Consequently, in this study, the widely used IMRT, VMAT, and HT were initially compared based on dosimetric and radiobiological evaluation. We sought to suggest a clear consensus on a preferred technique for EC in different location and generate better radiotherapeutic plans to guide clinical decision making.

Various DVH parameters were commonly used to evaluate treatment plan in clinical practice (36–38). However, the radiobiological analysis in view of the DVH curve as a whole might play a more critical role in determining the overall quality of treatment plan. Numerous studies indicated radiobiological models (28), which could immediately reflect tumor local control and OARs complications, should be regarded as an important consideration when selecting the optimal RT technique (11, 17). To date, no published articles have compared the IMRT, VMAT, and HT plans of EC using radiobiological evaluation. Therefore, the radiobiological parameters such as TCP and NTCP were calculated in this study. Our results undoubtedly extended and enlarged recent studies, and provided a new perspective for making clinical decisions. Integrating dosimetric and radiobiological parameters to evaluate treatment plan was more comprehensive and rational, especially in some cases yielding the analogical dosimetric metrics. As was reported in our study, IMRT, VMAT, and HT provided similar levels of dosimetry in lower thoracic EC, but radiobiological performance supported that VMAT was a better choice because of more superior TCP and lower NTCP of lungs and liver compared with IMRT and HT. Additionally, the radiobiological analysis was determined on the used models and parameters. In order to strengthen the reliability of our data, three independent predicting models were utilized in our study. Interestingly, we observed the similar trends of TCP and NTCP, and then further confirmed our results reliable. Taken together, we strongly suggested radiobiological evaluation should be widely adopted in clinical to cover the deficiency of dosimetric analysis.

Analysis of PTV coverage and dose homogeneity for IMRT, VMAT, and HT displayed different trends depending on tumor position. Wang et al. (37) compared the target coverage and dose distributions of HT, VMAT, and IMRT for locally advanced EC with SIB technique. HT proved to be superior to VMAT and IMRT in terms of CI and HI. Unfortunately, the study did not take the EC position into consideration. This was inappropriate and a limitation. As we reported, HT could yield the best CI and HI only in cervical EC but not in other positions. Another study was conducted to compare IMRT, HT, and VMAT for middle and distal EC (38). Being similar to this study, we also supported that IMRT was a preferred option for treating EC with large lungs volume involvement, since it could evidently reduce OARs dose with superior PTV coverage. Besides, our further analysis demonstrated that HT was a good option with little lung and heart involvement as it achieved excellent dose conformity and uniformity. VMAT was relatively appropriate with little thoracic involvement but surrounded by heart and liver. Although our findings were supported by previous evidences in a way, the hot topic which was the optimal RT technique for treating EC was still no consensus. Yin et al. performed a study to demonstrate that VMAT proved to be slightly better than IMRT in terms of target dose distributions for EC of all locations, and had equivalent or better OARs dose sparing and lower NTCP of lungs and heart (11). But we draw similar conclusions only in lower thoracic EC. Zhang et al. performed dosimetric analysis of TomoDirect (TD), HT, VMAT, and IMRT plans in upper thoracic EC, reporting that HT was considered as a good option with splendidly homogeneous and highly conformal dose distributions (36). However, IMRT rather than HT in our study provided superior CI and HI in upper thoracic EC. Analyzing the reasons leading to the controversial results, it may be attributed to the different numbers and angles of radiation fields, the experience of RT physicist and so on. More studies will be needed to support our results.

For carcinoma of the esophagus, radiation pneumonitis (RP) has been regarded as one of the most challenging toxicities. Numerous studies demonstrated the relationship between dosimetric parameters and RP, including MLD, V20, V13, V10, and V5 of lungs, in which V20 and MLD were strongly predictors (39–42). Tonison et al. (43) indicated that limiting the V20 to 23% or lower could keep the risk for grade≥2 RP below 10% for patients with EC who received chemoradiotherapy. Kwa et al. conducted a study of 540 patients irradiated for thoracic malignancy and proved the calculated risk of grade≥2 RP was 43, 18, and 11% for the MLD of 24–36, 16–24, and 8–16 Gy, respectively (44). The Quantitative Analysis of Normal Tissue Effects in the Clinic (QUANTEC) guidelines suggested if limiting the risk of RP ≤ 20%, it was prudent to restrict V20 ≤ 30–35%, and MLD ≤ 20–23 Gy with conventional fractionation (40). To reduce the incidence of RP in our study, V20 and MLD of lungs in IMRT, VMAT, and HT were strictly controlled in accordance with dosimetric requirements, and were not significantly different in the three plans for EC of all locations (all *p* > 0.05). Apart from V20 and MLD, emerging data suggested that percentage of lung volume receiving lower doses might be associated with RP. Emami et al. (29) pointed out that lungs V5 < 42% was related to the 5% incidence of symptomatic pneumonitis. While, V5 of lungs<42% was only achieved in cervical EC in this study. The increased low-dose volume of lungs should deserve more attention especially in EC cases with large thoracic involvement. In order to sharply reduce low-dose distribution of lungs, the fan-shaped virtual blocks in HT and the avoidance sectors in VMAT were used when we designed RT plans. Martin et al. (38) found that V10 and V15 of lungs in VMAT with two arcs were distinctly reduced than those in IMRT and HT for middle and distal EC. But these improvements came at the cost of higher dose to the heart. This prompted that if it was impossible to achieve all OARs dose constraints at the same time, how to make appropriate clinical decision for sparing each OAR was an urgent problem to be solved. Besides, according to our further analysis, it was clear that the target coverage and conformity for tumor and the dose constraints for normal organs should be well-managed in a tradeoff manner. In short, the better target coverage might lead to the more doses to OARs. The reduced dose of OARs had to come at the cost of the superior target coverage in some cases. How to solve these tough difficulties with compromise, clinician needed to comprehensively consider multiple factors.

Another hot topic was that radiation to the chest malignancy could exert long-term cardiac morbidities and mortality. Darby et al. (45) reported the rates of major coronary events increased linearly with the MHD by 7.4% per gray with no apparent threshold. V30 and V50 of heart have also been regarded as vital predictors for cardiac toxicities (46, 47). Up-dated evidences supported that the dose to heart was not only related to itself toxicity, but the combined dose to the heart and lung might have a synergistic effect on the increased risk of RP (48, 49). Thus, it was quite necessary to minimize dose to heart as low as possible. As the QUANTEC guidelines suggested, the long-term cardiac mortality could reduce <1% with <10% of heart V25. Although V30, V40, V50, and MHD of the heart were clinically acceptable in our study, it was really difficult to guarantee that heart V25 was <10% in particular for the middle and distal EC. Fortunately, we surprisedly found IMRT proved to be superior over VMAT and HT in terms of heart sparing for middle thoracic EC. It was time to pay close attention to adverse cardiac events for distal EC, because no present used RT technique could effectively reduce heart dose.

We needed to discuss the limitations of our study at this point. Firstly, because of a single-center and small sample size study, many confounding factors might affect the results. Our conclusions will be further confirmed. Secondly, more clinical trials investigating the local-regional control, overall survival and toxicity of different RT techniques for EC patients will be needed and aid the selection of the best possible treatment planning technique. Thirdly, with the advances in proton therapy (PT), it may be widely used in EC in future. However, no treatment plans of PT were generated in our study because of our institute not yet carrying out any related service. The dosimetry and radiobiology between PT and photon therapy will be compared in further study. But even so, given a solid based analysis, our study provided a new insight into better understanding of IMRT, VMAT, and HT plans characteristics in EC of different anatomical parts and aimed to guide clinical strategy.

## Conclusions

We initially suggested that EC of different location should be treated by different RT techniques. Moreover, radiobiological parameters such as TCP and NTCP should be widely used to evaluate treatment plans not only depending on dosimetric analysis. Overall, HT was a good option for cervical EC with complex target conformity but little lungs and heart involvement since it achieved superior dose conformity and uniformity. Due to potentially improving tumor control and reducing heart dose with acceptable lungs sparing, IMRT was a preferred choice for upper and middle thoracic EC with large lungs involvement. VMAT could ameliorate therapeutic ratio and lower lungs and liver toxicity, which was beneficial for lower thoracic EC with little thoracic involvement but being closer to heart and liver. Depending on the primary tumor location to choose optimal technique for EC will be warranted.

## Data Availability

All data generated or analyzed during this study are included in this published article.

## Ethics Statement

This study was approved by Ethics Committee of Shandong Cancer Hospital. Each patient gave written informed consent in accordance with the Declaration of Helsinki.

## Author Contributions

LW designed the study, generated the radiotherapy treatment plans, carried out data collection, statistical analysis, and wrote the initial draft. CQL, XS, and DS helped to generate the radiotherapy treatment plans. LP and YL contributed to data collection. XM participated in the design of the study. CML contributed to the statistical analysis. JL and JY supervised the research. All authors read and approved the final version of this manuscript.

### Conflict of Interest Statement

The authors declare that the research was conducted in the absence of any commercial or financial relationships that could be construed as a potential conflict of interest.

## Supplementary Material

Supplementary Materials Treatment plans of a patient with cervical (Image 1_v1), upper (Image 2_v1), middle (Image 3_v1), and lower (Image 4_v1) thoracic esophageal cancer.
