# Supplementary figures and images for: Dosimetric and Radiobiological Comparison of External Beam Radiotherapy Using Simultaneous Integrated Boost Technique for Esophageal Cancer in Different Location
Source: Front Oncol. 2019 Jul 25;9:674. doi: 10.3389/fonc.2019.00674 (PMC6669560; doi:10.3389/fonc.2019.00674)

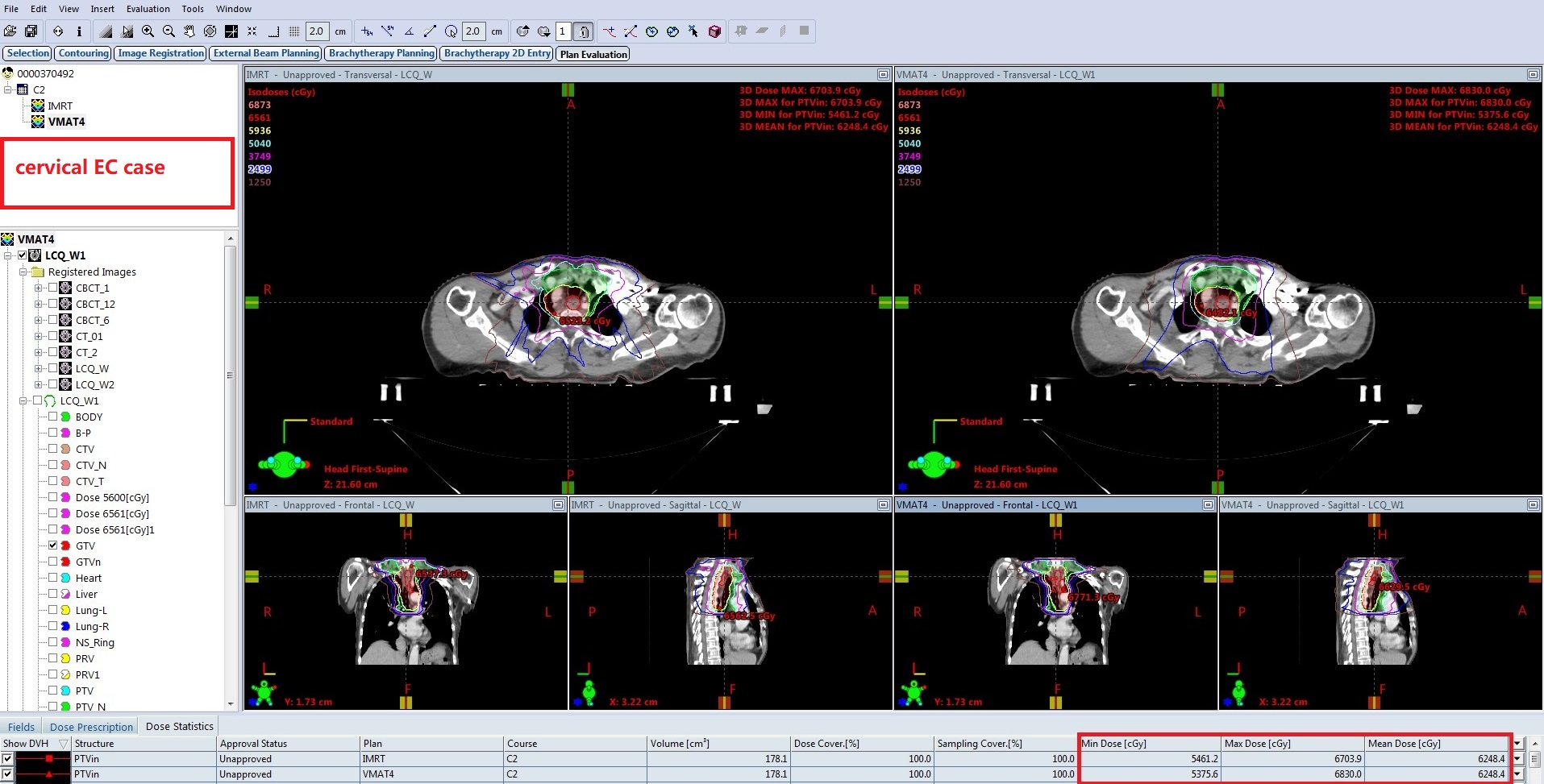

Supplement: Supplementary Materials — Treatment plans of a patient with cervical (Image 1_v1), upper (Image 2_v1), middle (Image 3_v1), and lower (Image 4_v1) thoracic esophageal cancer. [file Image_1.JPEG]

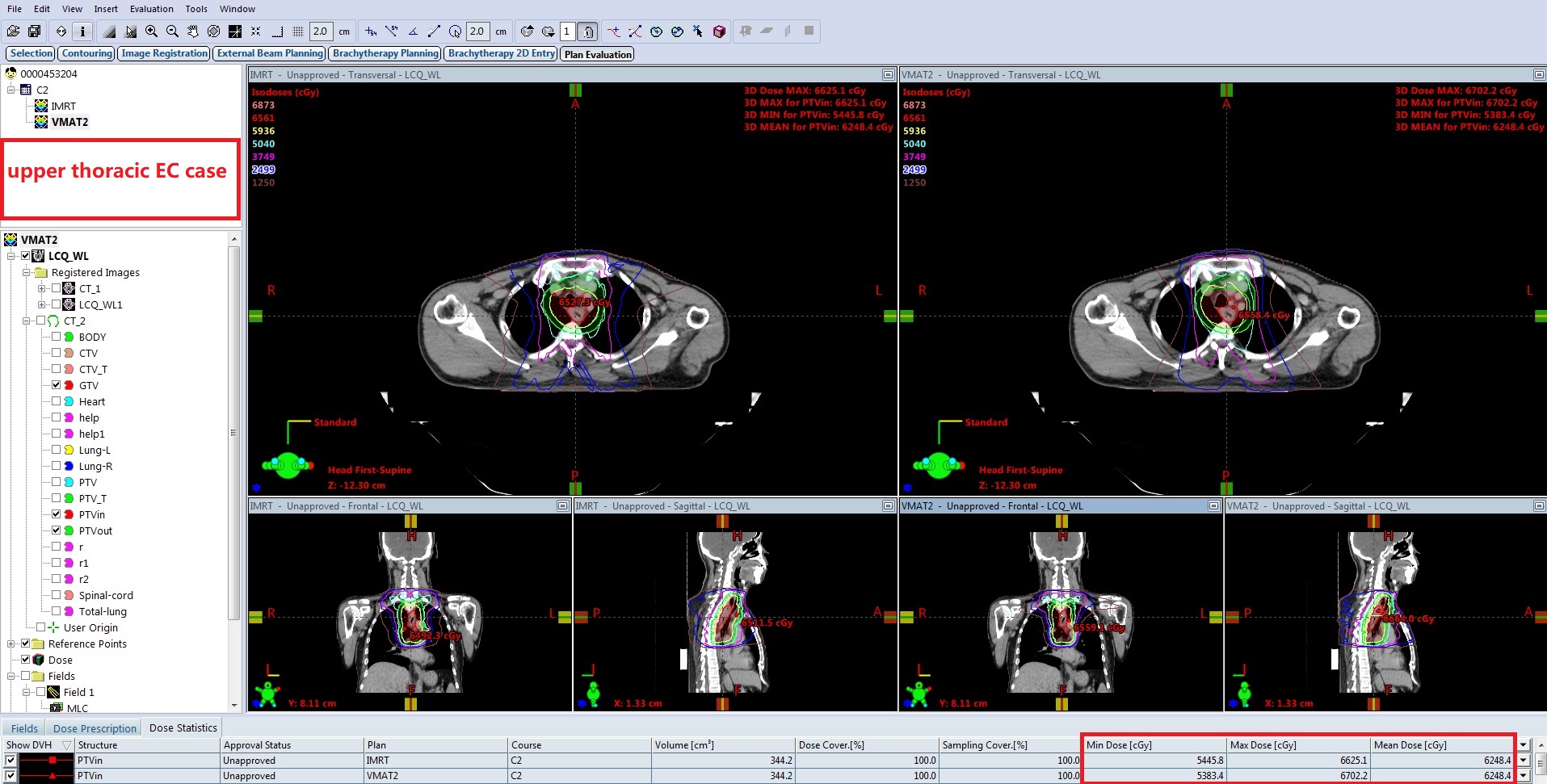

Supplement: Supplementary file 2 [file Image_2.JPEG]

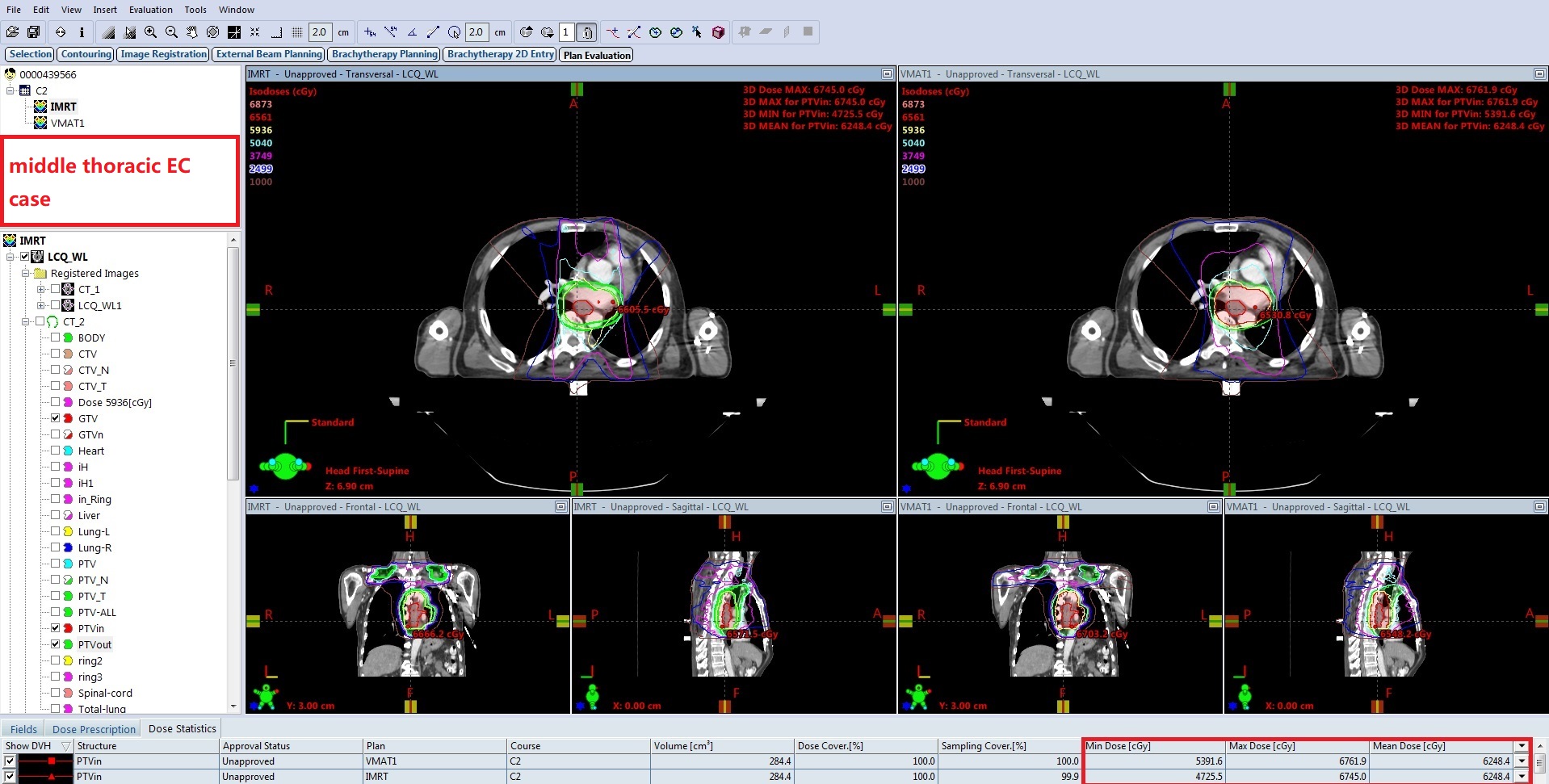

Supplement: Supplementary file 3 [file Image_3.JPEG]

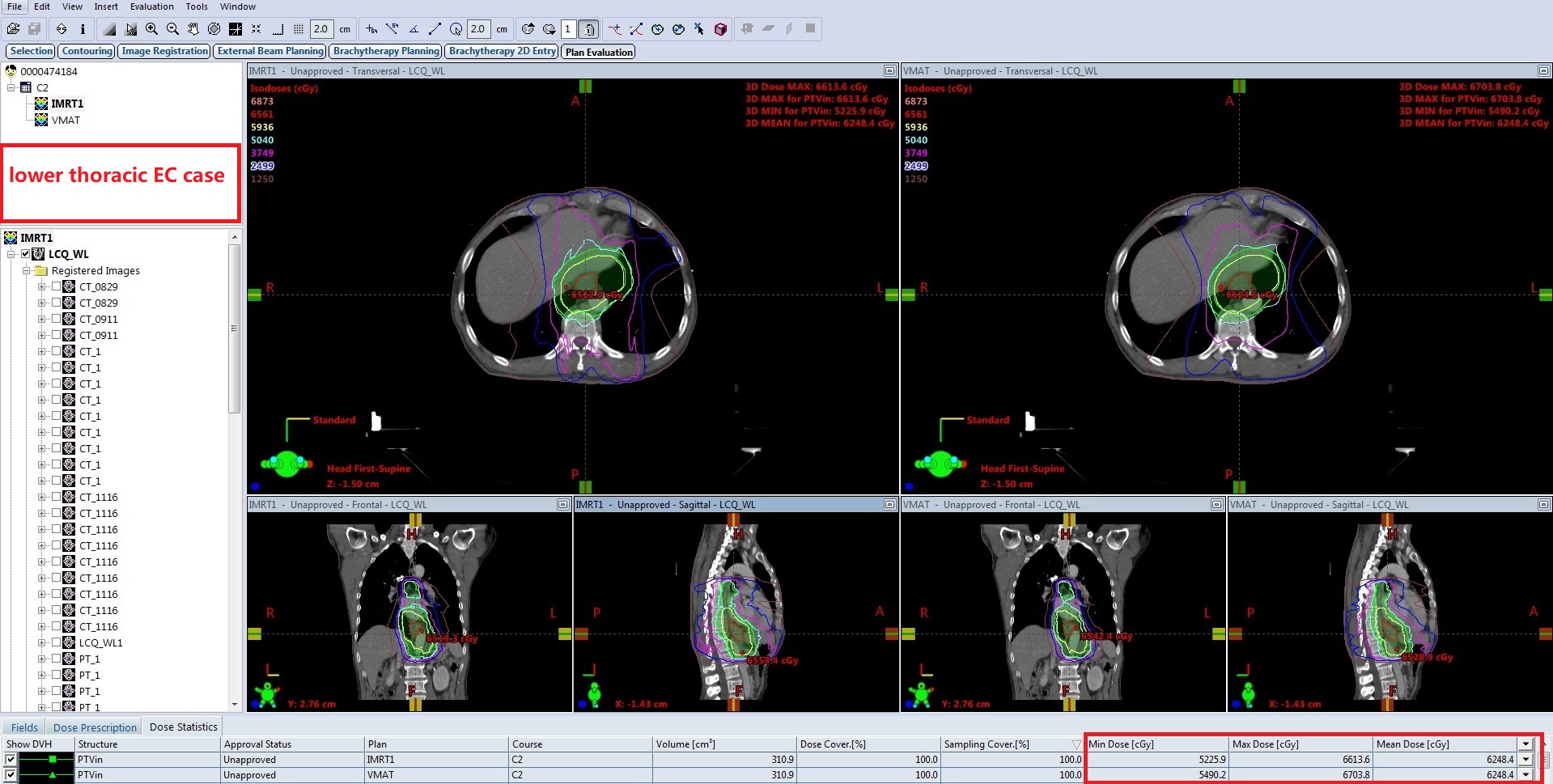

Supplement: Supplementary file 4 [file Image_4.JPEG]
